# Plant Resistance Inducers Affect Multiple Epidemiological Components of *Plasmopara viticola* on Grapevine Leaves

**DOI:** 10.3390/plants12162938

**Published:** 2023-08-14

**Authors:** Othmane Taibi, Irene Salotti, Vittorio Rossi

**Affiliations:** Department of Sustainable Crop Production, Università Cattolica del Sacro Cuore, Via Emilia Parmense, 84, 29122 Piacenza, Italy; othmane.taibi@unicatt.it (O.T.); irene.salotti1@unicatt.it (I.S.)

**Keywords:** cerevisane, Cos-Oga, fosytel-Al, laminarin, potassium phosphonate, *Pythium oligandrum*

## Abstract

Plant resistance inducers (PRIs) harbor promising potential for use in downy mildew (DM) control in viticulture. Here, the effects of six commercial PRIs on some epidemiological components of *Plasmopara viticola* (Pv) on grapevine leaves were studied over 3 years. Disease severity, mycelial colonization of leaf tissue, sporulation severity, production of sporangia on affected leaves, and per unit of DM lesion were evaluated by inoculating the leaves of PRI-treated plants at 1, 3, 6, 12, and 19 days after treatment (DAT). Laminarin, potassium phosphonate (PHO), and fosetyl-aluminium (FOS) were the most effective in reducing disease severity as well as the Pv DNA concentration of DM lesions on leaves treated and inoculated at 1 and 3 DAT; PHO and FOS also showed long-lasting effects on leaves established after treatment (inoculations at 6 to 19 DAT). PRIs also prevented the sporulation of Pv on lesions; all the PRI-treated leaves produced fewer sporangia than the nontreated control, especially in PHO-, FOS-, and cerevisane-treated leaves (>75% reduction). These results illustrate the broader and longer effect of PRIs on DM epidemics. The findings open up new perspectives for using PRIs in a defense program based on single, timely, and preventative field interventions.

## 1. Introduction

*Plasmopara viticola* (Berk. M. A. Curtis) Berl. & De Toni, the causal agent of grapevine downy mildew (DM), is a biotrophic Oomycete responsible for severe losses in both the yield and quality of wine grapes. However, DM management still largely relies on the use of synthetic and copper-based fungicides [1], which have limitations and are problematic given their negative impacts on the environment and human health [2]. Alternative products have been tried in recent years, and others are in development for achieving more sustainable and safe agricultural operations. Among the alternatives to fungicides, plant resistance inducers (PRIs) seem promising [3].

Induction of host resistance involves molecular interactions between *Vitis vinifera*, *P. viticola,* and the plant immunity system [4]. Plant pattern-triggered immunity (PTI), effector-triggered immunity (ETI), and the residual level of resistance qualified as basal resistance together constitute a plant’s innate immunity against biotrophic pathogens [5,6]. Briefly, when challenged, the evolved immunity memory, due to the priming process, stimulates or potentiates the deployment of inducible resistance (IR) [7]. The entire process leads to the development of systemic resistance (SAR) and/or induced resistance (ISR) [3,4]. These mechanisms are regulated by various phytohormones, such as salicylic acid, jasmonic acid, ethylene, and abscisic acid [8,9,10].

In viticulture, several compounds are known for their ability to induce resistance in grapevines against biotrophic pathogens, particularly *P. viticola* and *Erysiphe necator*, which have been reviewed by [3,4,11,12,13]. Some of these products have a natural origin; notable examples include laminarin (a beta-glucan extracted from the brown alga *Laminaria digitata*) and its sulfate derivatives [14,15]; chitosan (produced by the alkaline deacetylation of chitin obtained from insects, the exoskeleton of crustaceans, and fungal cell walls [16]; the mixture of chito-oligosaccharides (chitosan fragments) and oligo-galacturonides (pectin fragments from plant cell walls) known as Cos-Oga [17]; and cerevisane (extracted from cell walls of *Saccharomyces cerevisiae*) [18]. Other products consist of living microorganisms, such as *Pythium oligandrum* [19,20], while others are chemicals, such as benzothiadiazole, acibenzolar-S-methyl, fosetyl-Al [21], and potassium phosphonate, which also can directly inhibit the pathogen [15,22]. Recently, volatile organic compounds (VOCs) [23], in addition to beneficial microorganisms, such as the non-pathogenic *Trichoderma harzianum* T39 and the bacterium *Pseudomonas fluorescens* PTA-CT2 [24], as well as bioceramic silicon nitride (Si_3_N_4_) [25], RNA interference (RNAi) [26], and a grapevine by-product extract [27] have all been investigated for their capacity to induce plant resistance in grapevines. 

Several studies have elucidated the physiological changes that happen in the grape tissue (mostly for cell suspensions, leaves, and berries) after the application of resistance inducers and subsequent artificial inoculation with *P. viticola* [12,26,27,28,29]. Most of these studies focused on the plant defense reaction and signaling events that occur upon the host’s recognition of pathogenic patterns. These result in reactive oxygen species (ROS) accumulation, ions fluxes, mitogen-activated protein kinase (MAPK), cell-wall reinforcement, and the expression of pathogenesis-related proteins (PR), which elucidate the PTI response [8,9] as well as the synthesis of fungi-toxic stilbenes and subsequent hypersensitive reaction (HR) and programmed cell death, which underpin the highly specific ETI reaction [10,30,31]. Unlike numerous studies done under controlled conditions, surprisingly few have investigated the use of resistance inducers under vineyard conditions [32,33,34,35]. Yet, few of these products showed acceptable efficacy against *P. viticola* when tested in the field. Delaunois et al. [4] discussed these differences in effectiveness and related them to PRIs’ broader mode of action, levels of disease pressure, vine stress conditions in the field, and local weather conditions. Moreover, applied research on how to integrate PRIs into practical disease management is still lacking, and additional research aimed at a holistic understanding is needed [3]. 

To the best of our knowledge, no field study has yet been conducted to clarify the effects of resistant inducers on the epidemiological components of *P. viticola*. Epidemiological components refer to an infection monocycle and include the following aspects: infection efficiency of sporangia on leaves (the number of sporangia vs. the number of DM lesions; i.e., the typical chlorotic ‘oil’ spot), length of incubation (the elapsed time between infection and lesion onset), size of DM lesions, length of the latency period (the elapsed time between infection and onset of sporulation on the lesions), production of sporangia on the lesions, and duration of the infectious period (how long a lesion continues to produce sporangia) [36]. These monocycle components are of paramount importance because they modulate the disease progress of polycyclic pathogens such as *P. viticola*, in which several infection cycles develop during the grape-growing season [37,38]. For instance, the slowed progression of DM in partially resistant grapevine genotypes [39] originates from the joint effect of resistance acting upon multiple epidemiological components [40]. 

In this work, we investigated several commercial inducers of plant resistance having different origins (chemical, natural, and microbial) for their impact on key epidemiological components of *P. viticola* on grapevine leaves. Through artificial inoculations and monocyclic experiments, we studied the infection efficiency of sporangia (expressed as disease severity on the adaxial leaf blade), the mycelial colonization of leaf tissue (as the concentration of *P. viticola* DNA in both leaves and DM lesions), the size of the sporulating area (as sporulation severity in the abaxial leaf blade), and the production of sporangia on affected leaves and per unit area of DM lesions. Our study comprehensively addressed the efficacy of resistance inducers for preventing DM infection and considered possible effects on preventing the further spread of the disease via the production of secondary sporangia on affected leaves.

## 2. Results

Disease data for the leaves of nontreated plants following their artificial inoculation with *P. viticola* sporangia are shown in Table 1. Disease severity was higher in Experiment 1 (average ± SE: 34.2% ± 3.12) than in Experiment 2 (3.9% ± 0.83) or Experiment 3 (9.8% ± 1.21), so was the length of the latent period (averaging 10.0, 18.6, and 15.6 days, respectively, between inoculation and sporulation of DM lesions). In Experiment 1, treatments were applied at GS73, and the weather conditions were mild (average temperature between *P. viticola* inoculation and sporulation onset on NT leaves was 20.4 °C, relative humidity was 67.6%, hours of leaf wetness were 35.6 and rainfall was 38.1 mm). In both Experiment 2 and 3, treatments were respectively implemented at GS79 and GS75 when temperatures were higher (25.1 and 24.9 °C, respectively) and humidity lower (60.5 and 54.4%, respectively). 

The analysis of covariance (ANCOVA) detected a significant (*p* < 0.001) effect of all the considered factors and interactions. Results of the three-year experiments are shown with a focus on the effect of treatment and that of the interaction term ‘treatment × DAT’. The significant interaction between ‘experiment × treatment × DAT’ was not considered because it accounted for less than 10% of the total variance and because we were more interested in the average response to treatments rather than the variation among years (experiments). 

The ANCOVA also revealed a highly significant effect of the covariate (*p* < 0.001), i.e., leaf size, with respect to disease severity, sporulation severity, sporangia produced per leaf, and Pv DNA concentration in the DM lesions. By contrast, no significant effect of leaf size was found for sporangia produced per cm^2^ lesion, or for the Pv DNA concentration of the entire leaf. For each response variable, its average value was then covariate-adjusted. The relationships between leaf size and the different response variables are plotted in Figure S1 for the nontreated control (see Supplementary Materials). Both disease and sporulation severity, as well as the sporangia produced per leaf, all increased significantly (*p* < 0.001) with the leaf size at the time of disease assessment. Since all the inoculated leaves were similar in age and size at the time of their *P. viticola* inoculation, these results indicated that with greater leaf expansion, there is greater susceptibility to DM. Conversely, the Pv DNA concentration in DM lesions decreased with increasing leaf size, indicating a lower mycelium density in the affected leaf tissue.

### 2.1. Effects of Resistance Inducers on Disease Severity and P. viticola DNA Concentration in Leaves

**Figure 1:**
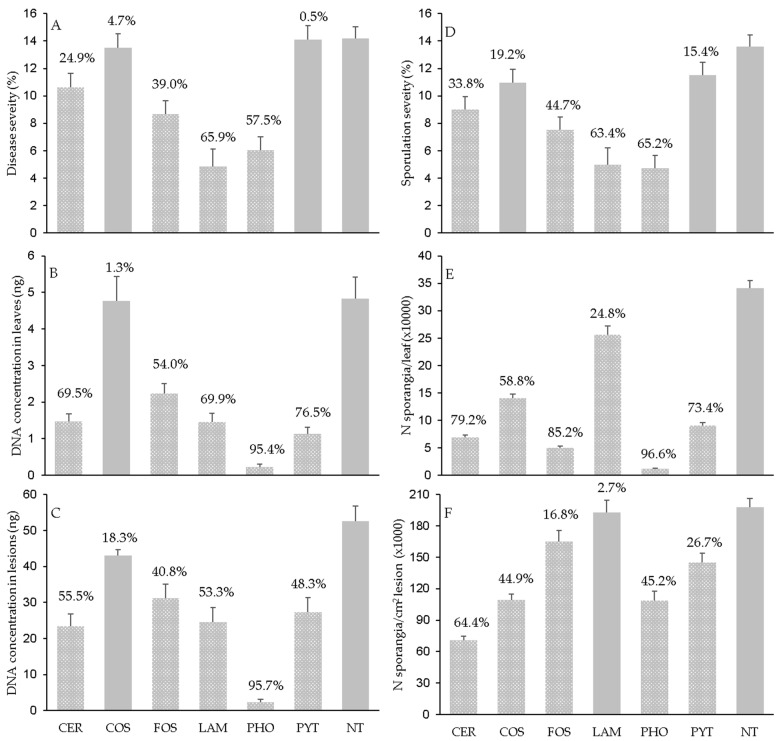
Assessments of downy mildew (**A**–**F**) in grapevine leaves collected from plants that were treated with the plant resistance inducers in Table 3 or left untreated (NT) and then inoculated with *Plasmopara viticola* (Pv). Bars are means (of N = 3 experiments, five inoculation time points, 15 leaves per time point) whose whiskers are standard errors. Bars are solid gray when averages did not differ significantly from NT, at *p* ≤ 0.05; the percentage value above each bar shows the effect size (i.e., reduction in comparison to NT).

**Figure 2:**
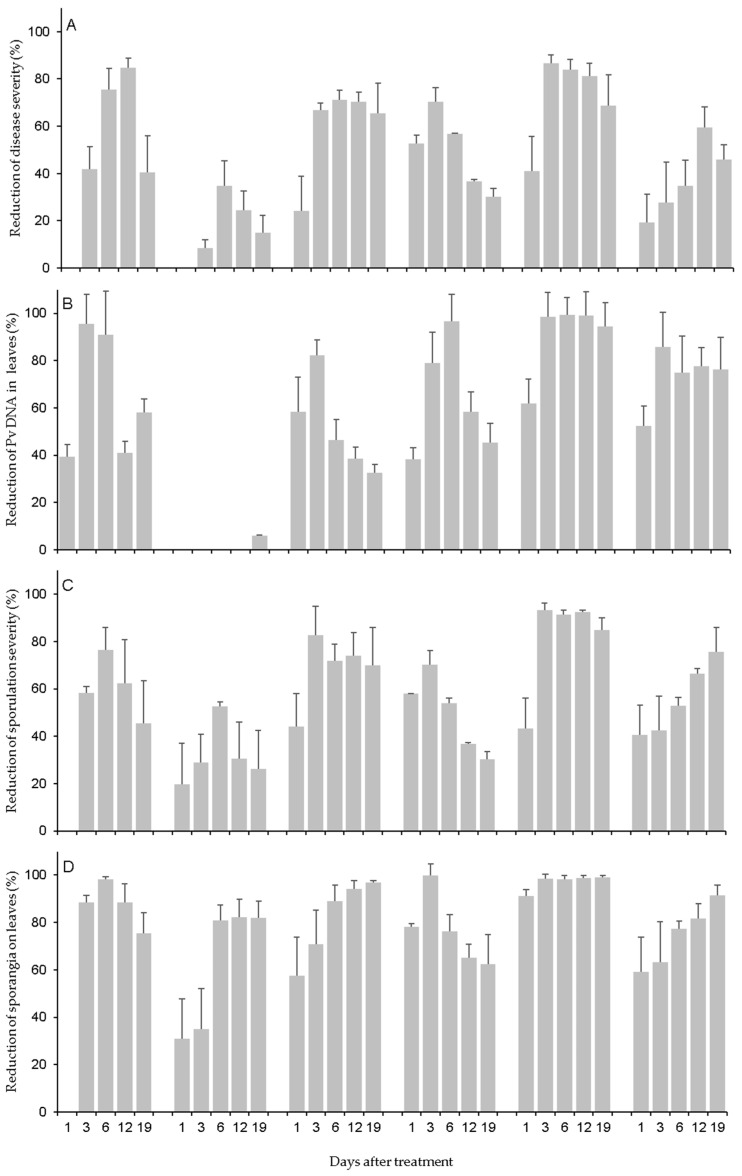
Assessments of downy mildew (**A**–**D**) in grapevine leaves collected from plants that were treated with the plant resistance inducers in Table 3 and then inoculated with *Plasmopara viticola* (Pv) at 1 to 19 days after treatment (DAT). Bars are means (of N = 3 experiments, 15 leaves per DAT) whose whiskers are standard errors. Data are expressed as the percentage reduction in comparison to the nontreated leaves.

Disease severity on leaves (Figure 1A) was higher in NT, PYT, and COS (14.2% ± 0.88, 14.1% ± 1.0, and 13.5% ± 1.00 of affected leaf area, respectively) than the other products, with LAM (4.8% ± 1.28) and PHO (6.0% ± 0.99) showing the least severity, being respectively reduced by 65.9% and 57.5% relative to NT. Because of the ‘treatment × DAT’ interaction, the applied products reduced the disease severity at 1 DAT by 24.2% (for FOS) to 52.8% (for LAM) when compared to NT, excluding CER and COS that did not cause a reduction in disease severity when leaves were inoculated at 1 DAT (Figure 2A). The treatments further reduced disease severity when leaves were inoculated until 6 or 12 DAT, but this depended on the product; afterward, the reduction was lower in response to CER, COS, LAM, and PYT but went unchanged for FOS and PHO (Figure 2A). 

The Pv DNA concentration in leaves (Figure 1B) reflected, in part, the pattern in the disease severity data (Figure 1A), with a correlation coefficient for the pooled data being *r* = 0.761 (*p* ≤ 0.001). Correlation coefficients, however, were slightly higher for the leaves collected from nontreated (*r* = 0.793) than treated (*r* = 0.748) plants (Table 2), indicating that the treatments weakened the associations. Both NT and COS, which had similar disease severities, also had similar concentrations of Pv DNA, but this was not the case for PYT, for which the DNA concentration in leaves was lower than in NT (with a 76.5% reduction) (Figure 1B). This discrepancy was caused by a lower DNA concentration in DM lesion for PYT than for NT (27.3 and 43.0 ng of DNA, respectively). Notably, the DNA concentrations in leaves and DM lesions were both very low for PHO (Figure 2C). Overall, the DNA data indicated that most of the resistance inducers were capable of reducing the mycelium growth of *P. viticola* within the visible DM lesions by 55.5% (for CER) to 95.7% (for PHO).

**Table 2:** Pearson correlation coefficients between different assessments of downy mildew in grapevine leaves collected from plants treated with the plant resistance inducers in Table 3 or left untreated and then inoculated with *Plasmopara viticola* (Pv).

Dataset	Disease Assessments	Sporulation Severity (%)	Pv DNA Concentration in Leaves	Pv DNA Concentration in Lesions	No. of Sporangia/Leaf	No. of Sporangia/cm^2^ Lesion
All data	Disease severity (%)	0.942 *	0.761 *	0.294 *	—	—
	Sporulation severity (%)		—	—	0.593 *	–0.051
	Pv DNA concentration/leaf			—	—	—
	No. of sporangia/leaf				—	0.770 *
Untreated	Disease severity (%)	0.966 *	0.793 *	0.396 *	—	—
	Sporulation severity (%)		—	—	0.555 *	–0.065
	Pv DNA concentration/leaf			—	—	—
	N sporangia/leaf				—	0.808 *
Treated	Disease severity (%)	0.933 **	0.748 *	0.271 *	—	—
	Sporulation severity (%)		—	—	0.546 *	–0.116 *
	Pv DNA concentration/leaf			—	—	—
	No. of sporangia/leaf				—	0.762 *

* *p* ≤ 0.01; ** *p* ≤ 0.001.

**Table 3:** Plant resistance inducers tested in the experiments and their application dose.

Active Ingredient	Acronym	Trade Name and Manufacturer	Reference N°	Concentration	Dose
Cerevisane	CER	ROMEO, SUMITOMO Chemical Italia S.r.l., Milan Italy	17,058	94.1%	0.25 kg/ha
COS-OGA	COS	IBISCO, GOWAN Italia S.r.l., Faenza Italy	16,509	12.5 g/L	2–3 L/ha
Fosetyl-Aluminium	FOS	ALIETTE, BAYER Italia S.p.a., Milan Italy	4710	80%	2.5 kg/ha
Laminarin	LAM	VACCIPLANT, UPL Italia S.r.l., Cesena Italy	15,831	45 g/L	1.5 L/ha
Potassium phosphonate	PHO	CENTURY, BASF S.p.a., Cesano Maderno Italy	16,657	755 g/L	2 L/ha
*Pythium oligandrum*	PHY	POLYVERSUM, GOWAN Italia S.r.l., Faenza Italy	16,654	1 × 10^6^ CFU/g	0.3 kg/ha

Regarding disease severity, the Pv DNA concentration in leaves was evidently affected by the time elapsed between treatment and *P. viticola* inoculation (Figure 2B). For all six tested products except COS, there was a reduction in the DNA concentration vis-à-vis NT starting from 1 DAT. This reduction strengthened from 3 to 6 DAT but then gradually decreased over the later DAT, except in the case of PHO, as well as LAM, where it remained relatively stable over time until 19 DAT. For COS, as mentioned already, there was no reduction in Pv DNA when compared to NT. 

**Figure 3:**
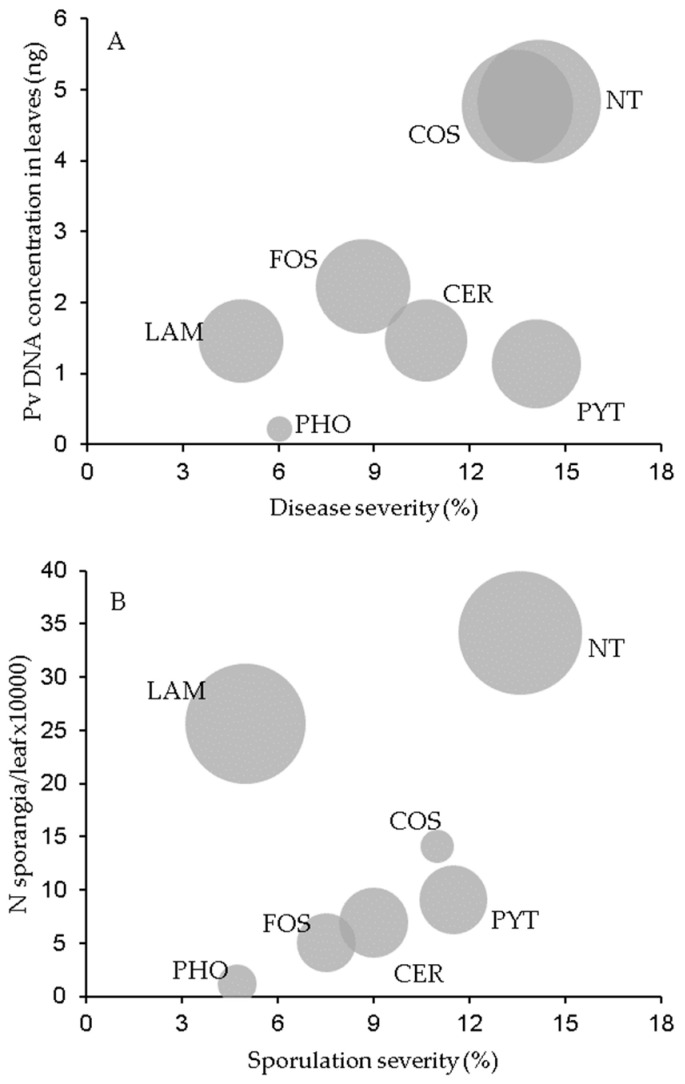
Bubble chart showing the relationship between downy mildew severity and the content of *Plasmopara viticola* (Pv) DNA in grapevine leaves (**A**) and that between sporulation severity and the production of sporangia on leaves (**B**). Grapevine leaves were collected from plants treated with the plant resistance inducers in Table 3 or left untreated (NT) and then inoculated with *P. viticola*. Each bubble corresponds to the mean of N = 3 experiments, with five inoculation time points and 15 leaves per time point.

The effects of resistance inducers on the disease severity and DNA concentration in leaves and DM lesions are summarized in Figure 3A as a bubble chart. The three disease assessments were judged to be low in terms of leaves collected from plants treated with PHO and high for those treated with COS. Leaves treated with LAM exhibited low disease severity but an intermediate overall DNA concentration, given that the DNA in their lesions was high (compare the bubble sizes in Figure 3A). Conversely, PYT-treated leaves were distinguished by a high disease severity but a low overall DNA concentration.

### 2.2. Effects of Resistance Inducers on the Sporulation Severity and Production of P. viticola Sporangia on Leaves

The sporulation severity of grapevine leaves was closely correlated with their disease severity (*r* = 0.942; *p* < 0.001) (Table 2). The linear regression had an intercept (−0.241 ± 0.160) not significantly different from zero (*p* = 0.134) and a slope (0.888 ± 0.008) significantly different from 1 (*p* < 0.001), with R^2^_adj_ = 0.899. Testing for the equality of slopes for the single products and NT gave a log-likelihood value close to zero, indicating that the relationship between disease and sporulation severity was not affected by treatments. 

Because of the close correlation between disease severity and sporulation severity, the latter was higher for leaves from NT plants (13.6% ± 0.83 of leaf area with visible sporulation) and from those plants treated with PYT (11.5% ± 0.95) and COS (11. 0% ± 0.95) than for the other resistance inducers, with LAM and PHO showing a reduction in sporulation severity of >60% relative to NT (Figure 1C). The dynamics of reduction in sporulation severity for leaves inoculated at different DATs (Figure 2C) showed similar patterns for disease severity (Figure 2A). 

Sporulation severity was significantly correlated with the number of *P. viticola* sporangia produced per leaf (*p* < 0.001), but the correlation was weak (*r* = 0.593 for all leaves; Table 2). Leaves from plants treated with PYT and COS, which had similar sporulation severity to NT (Figure 1D), produced 9.06 ± 0.52 and 14.07 ± 0.77 sporangia (×10^3^), respectively, corresponding to 73.4% and 58.3% fewer sporangia than for NT (34.12 ± 1.39 sporangia × 10^3^) (Figure 1E). Conversely, leaves from LAM-treated plants produced more sporangia (25.66 ± 1.58 sporangia × 10^3^) than those treated with COS or PYT, even though the latter two led to higher sporulation severity. Sporangia were least abundant on PHO-treated leaves (1.17 ± 0.10 sporangia × 10^3^), amounting to a 96.6% reduction relative to NT (Figure 1E). The lower production of sporangia on leaves was closely related to a lower sporulation per unit (cm^2^) of DM-affected tissue, with *r* = 0.770 (*p* < 0.001) for all data pooled. In addition to LAM, the number of sporangia produced per cm^2^ of DM lesions was higher on the leaves from nontreated (198.1 ± 8.04 sporangia × 10^3^) than treated plants, the latter ranging from 70.5 ± 4.24 for CER to 164.8 ± 11.0 for FOS (Figure 1F). 

As found for sporulation severity, the production of sporangia on leaves was affected by the time elapsed between treatment and *P. viticola* inoculation (Figure 2D). For instance, LAM and PHO reduced the production of sporangia by 78.1% and 98.1%, respectively, at 1 DAT when compared to NT. Afterward, the reduction effect peaked at 99.7% at 3 DAT and was moderately lower at 6 to 19 DAT for LAM; by contrast, the reduction effect was always >98% for PHO.

The effect of the tested products on sporulation severity and the production of sporangia on leaves and in DM lesions are summarized in Figure 3B. Numbers of sporangia per leaf and per cm^2^ DM lesion (bubble value) following the treatments generally increased as sporulation severity increased, except in the case of LAM, for which sporulation was high despite a low sporulation severity (perhaps a consequence of diminished disease severity), and COS, for which the production of sporangia per DM lesion unit was low.

## 3. Discussion

Our experimental setting aimed at studying the effectiveness of PRIs over time following their application in the vineyard. These PRIs were applied to grapevines at the label dose for preventative control of DM, i.e., before infection by *P. viticola*, as suggested by both their manufacturers and good practices [41]. Afterward, infection was artificially induced by spraying a *P. viticola* sporangial suspension on single, unfolded leaves in the apical position of growing shoots; for this, we selected young, expanding leaves for artificial inoculation because they are highly susceptible to infection, unlike older leaves that show ontogenic resistance [42,43,44]. Another reason for inoculating the apical leaves of shoots is related to the relevance of protecting the growth of new plant tissues after treatment, especially in periods when shoots grow rapidly [45]. 

Artificial inoculations with *P. viticola* were carried out from 1 to 19 days after treatment (DAT) to study how long the protection provided by PRIs lasted following a sole application and whether that protection level differed among the DATs. In previous studies, PRI effectiveness was assessed based on disease severity on leaves following a single *P. viticola* inoculation made to plants that had been treated multiple times with PRIs [12,25,35,46,47], or those treated with PRIs after a pathogen inoculation [23]. Compared with such studies, our experimental approach better reflects the real conditions in a vineyard, where the time lag between treatment and infection is almost always unknown. 

Our experiments were conducted over 3 years to account for some variability due to weather conditions and grapevine phenology (GS73, GS75, and GS79 in the 3 years, respectively). Both factors contributed to differences found among years and DAT (15 *P. viticola* inoculations in total) in the length of latency period (i.e., the time between inoculation and sporulation on DM lesions) and the DM severity of nontreated plants’ leaves. Another source of variability in our experiments could be the defense status of the grapevines at the time of their treatment and *P. viticola* inoculation because, under natural conditions, plants are also primed by various biotic and abiotic stresses that can interfere with the efficacy of PRIs [4,48]. Overall, the large variability we observed made the results robust. 

We should note that, in our experiments, leaves inoculated at 1 to 3 DAT were already present on the grapevines when they received a PRI application, while leaves inoculated at 6 to 19 DAT were not because they formed post-treatment. Consequently, the PRI efficacy on *P. viticola* we observed at 1 to 3 DAT could have been caused by both direct effects of the active ingredient against the pathogen, if any, and induction of resistance; however, the efficacy at 6 to 19 DAT could only be caused by induction of resistance, since a direct effect may be unlikely. Resistance inducers have been studied particularly for their priming effect and the subsequent plant immunity response [11,15,31,46,49,50,51,52]. The effect of a given PRI can involve several pathways and mechanisms that lead to a SAR and/or an ISR, and it depends on a faster transcription of a few genes, the accumulation of antimicrobial molecules in the plant, and entails plant growth/defense trade-offs [3,10,11,50,53]. Some direct activity of PRIs against oomycetes has been documented, however [22,28,30,54,55]. 

In our study, laminarin (LAM), potassium phosphonate (PHO), and fosetyl-Aluminum (FOS) were the most effective in reducing the disease severity and Pv DNA concentration in DM lesions following *P. viticola* inoculation to leaves extant at the time of treatment (i.e., inoculations made at 1 and 3 DAT). In particular, PHO and FOS also had a long-lasting protective effect on those leaves established post-treatment (inoculations at 6 to 19 DAT). 

Laminarin is a water-soluble polysaccharide that consists of β-(1-3)-glucan derived from the brown alga *Laminaria digitata* [56]. It effectively reduced *P. viticola* infection through plant defense activation in leaf disk assays [49], under semi-controlled conditions [15,31,33,57], and in vineyard settings [58,59]. LAM triggers the expression of defense genes, callose deposition, and phenolic compounds’ accumulation, leading to HR-like cell death at the infection site [31,60]. In grapevine-cultured cells, LAM is also able to elicit calcium influx, alkalinization of the extracellular medium, an oxidative burst, induction of MAPK and PR genes, and the production of phytoalexins. In plants, LAM has been found to elicit the expression of certain PR genes, such as β-1,3-glucanase, chitinase, and serine-proteinase inhibitor (PIN) [15,61,62]. Similarly to the present findings, in previous studies, LAM-treated plants responded quickly [15,18,46,49]. This prompt reaction has been linked to strong hydrogen peroxide (H_2_O_2_) up-regulation Trouvelot, Varnier [31], [63], which could reduce stomatal opening and impair a pathogen’s penetration of the host [64]. A direct effect of LAM was found against *Zymoseptoria tritici* in wheat [65], but its role against *P. viticola* is controversial. Besrukow et al. [66] observed impaired zoospore motility, and Mestre, Arista [67] identified an endo-β-1,3-glucanases able to exert a direct effect against DM, whereas Gauthier, Trouvelot [49] and Steimetz, Trouvelot [63] reported no direct effect of LAM on *P. viticola*. Other studied natural products, however, can directly affect *P. viticola*. For instance, an unspecified plant extract had a strong direct effect on the release and motility of *P. viticola* zoospores, and other plant extracts were shown to impair these zoospores’ ability to reach stomata on the leaf surface [28,66,68,69]. The capability of LAM to directly impair *P. viticola* infection warrants further investigation. 

PHO is a salt of phosphonic acid, and FOS is a salt of ethyl phosphonic acid, both of which are transformed into phosphonates in the plant; accordingly, PHO and FOS share the same active ingredient [70]. Phosphonates get distributed in the plant via phloem and xylem tissues and can induce a plant defense reaction [47,54,71,72,73] through cell-wall reinforcement, ethylene biosynthesis, and phytoalexin accumulation [74]. Phosphonates, once introduced into the mycelium of *Phytophtora* species, were shown to directly suppress ATP and NAD synthesis and, consequently, stop pathogen growth and inhibit spore germination and penetration into host plants [75]. Phosphonic (phosphorous) acid, however, demonstrates high efficacy in the post- but not pre-infection control of *P. viticola* [21,71,76,77,78]. To our knowledge, our results provide the first empirical evidence for the prompt, effective, and long-lasting effectiveness of PHO against DM in preventative applications against *P. viticola*. 

Although CER was less effective than PHO, FOS, or LAM in reducing the DM severity, it exerted a similar effect to those in reducing the growth of *P. viticola* in DM lesions (in terms of their Pv DNA concentration). CER did not affect DM severity at 1 DAT, but it was increasingly effective at 3 to 12 DAT and then subsided at 19 DAT. CER is a yeast-derived PRI made of purified cellular walls of *Saccharomyces cerevisiae* strain LAS117 that is known to be effective in reducing DM [18] by mobilizing plant defense reactions. In a global transcriptome analysis of grapevine leaves sprayed at 1-week intervals, CER caused an increase in the expression of several genes related to defense responses to pathogens and other stresses, along with the down-regulation of genes involved in several processes related to plant growth and development. Mishko and Lutsky [46,79] observed that the application of *S. cerevisiae*—from which CER is extracted—enhanced the immune response in a DM-resistant grape variety and inducted the synthesis of phytoalexins in a susceptible variety. No direct effect of CER has been documented so far, unlike for some hydrolytic enzymes and VOCs volatile organic compounds produced by *S. cerevisiae* that demonstrated antifungal activity against *Colletotrichum acutatum* on citrus [80]. 

We found that COS and PYT were less effective in reducing the DM severity on leaves, in spite of PYT being able to lessen pathogen growth in the DM lesions. COS is composed of chitosan oligosaccharides (COS)–oligogalacturonides (OGA) complex that has been found to induce resistance against biotrophic pathogens in different crop plants [81,82,83] by activating the salicylic acid pathway [11,17]. As a mycoparasite of pathogenic fungi in the soil, PYT is able to degrade fungal cell wall polysaccharides and induce systemic resistance in plants [84,85,86]. These two products have been considered for their effectiveness in controlling powdery mildew (namely COS and PYT) and grey mold (namely PYT) in previous reports [17,19,20,34,51,84,85,86]. Our results here show that these PRIs, when used alone, can also influence *P. viticola* infection, albeit to a lesser extent than the other tested products, which is a novel finding to our best knowledge. 

In addition to the reduced DM severity and pathogen growth in DM lesions, as our results show, the investigated PRIs also prevented the sporulation of *P. viticola* on lesions. The number of sporangia produced on leaves was lower in all the PRI-treated leaves compared to the non-treated control, but especially in PHO-, FOS- and CER-treated leaves (with a >75% reduction). That *P. viticola* sporulation is lower in grapevine leaves of the varieties carrying R*pv* genes, which confer partial resistance to DM than in susceptible ones, has been documented by Bove and Rossi [40]. However, this is the first time such a reduction has been documented for leaves of susceptible grapevine varieties treated with PRIs. This is an important finding because it illustrates a broader and longer effect of PRIs on DM epidemic dynamics, which is not limited to the prevention of an infection event and the consequent reduction of disease severity. In epidemiological terms, reduced production of sporangia on lesions adversely affects the basic infection rate of the epidemic [87] and, thus, how the disease progresses over time [88]. Epidemiological components other than sporulation are altered in partially resistant grapevine varieties [40], namely, the infection frequency (i.e., the proportion of sporangia able to cause another infection) and lesion size (which together determine disease severity), as well as the latency period (i.e., the time between infection and the start of sporulation on lesions). Whether these components are also changed by PRIs remains to be investigated. 

From a practical standpoint, our study’s results open up new perspectives on how to use PRIs when devising a defense program against DM based on single, preventative interventions performed 1 to 3 days before *P. viticola* infection, depending on the product used. This may enable vineyard managers to modify or circumvent the current strategy for using PRIs, which is based on a calendar and repeated applications instead of the actual risk of infection posed at a site. Risk-based preventative application of PRIs is possible nowadays because of the rising use of decision support systems [89] in which process-based models provide a robust prediction of infection periods by *P. viticola* [90,91]. Hence, switching to a pre-infection usage of PRIs could lower the number of interventions required in a vineyard [92]. It may also decrease the metabolic cost for the plant targeted for protection. Such a cost can arise from the allocation of energy and metabolites [93] or physiological alterations—e.g., deposition of dormant signaling enzymes or modification to histones on defense gene promoters. [94]—associated with the priming process that shifts the plant into a primed state of defense in which it is conditioned for the super-activation of defenses against a pathogen challenge [95]. The metabolic cost of priming is counterbalanced by disease mitigation in the case of a potentiated plant defense response triggered by the pathogen [93]. Still, it is not necessarily the case that no pathogen challenge occurs following a PRI application, i.e., when PRIs are applied during infection-free periods. 

## 4. Materials and Methods

### 4.1. Vineyard Characteristics

The experiments were conducted from 2020 to 2022 in a vineyard of *V. vinifera* cv. Barbera that is highly susceptible to DM [96], at the Res Uvae experimental farm in Castell’Arquato (Piacenza, Italy; 44°51′26.031″ N, 9°51′21.779″ E). 

The vineyard, lying flat, was 15 years of age in 2020. It is trained using the simple Guyot system, with rows oriented North–South, with grapevines spaced 2.4 m between rows and 1.3 m apart along the row, for a plantation density of 3204 plants/ha. The vineyard was managed following the common practices of the grape-growing region, with the exception of fungicide treatments to control DM in the plot where the experiments were conducted. At the time of the experiments, there were no natural DM symptoms in the vineyard.

The hourly values of temperature (°C), relative humidity (%), precipitation (mm), leaf wetness (yes/no), and wind speed (m/s) were recorded by a weather station (iMetos^®^, Pessl Instruments, Weiz, Austria) located in the experimental vineyard.

### 4.2. Experimental Setting

Plots of five plants were set up at least 4 m from each other. The following treatments were assigned at the plot level according to a complete randomized design, with three replicate plots per treatment: nontreated control (NT), cerevisane, Cos-Oga, fosetyl-Al, laminarin, potassium phosphonate, and *Pythium oligandrum* (Table 3). These six products were applied using a battery-powered backpack sprayer (Volpi S.p.a., Casalromano, Italy) at a pressure of 4.5 bar, with a spraying volume of 4 hL/ha. Applications were made on 28 May 2020, 7 July 2021, and 1 June 2022, when plants were respectively at the growth stage (GS) 73, 79, and 75, according to Lorenz et al. [97].

### 4.3. Inoculation of Grapevine Leaves with P. viticola 

At 1, 3, 6, 12, and 19 days after treatment (DAT), 5 to 7 leaves were tagged per each of the three plots (for a total of 20 leaves per treatment per time point) from as many actively growing shoots. All tagged leaves were located at the 3rd or 4th position below the first unfolded leaf from the shoot apex; these leaves were in the expansion stage and thus highly susceptible to *P. viticola* [98]. Tagged leaves were inoculated by distributing a suspension of *P. viticola* sporangia (5 × 10^4^ sporangia/mL) on the abaxial leaf blade with a manual nebulizer. To ensure infection [99], all inoculations were carried out in the late afternoon, and the inoculated leaves were immediately enclosed in transparent polyethylene bags and then sealed with clothespins to maintain a saturated atmosphere; bags were removed the next morning, ca. 14 to 16 h post-inoculation. Since the growing shoots had a leaf emergence rate of a single leaf per ca. 3 days, the leaves inoculated at 1, and 3 DAT were directly sprayed with the products, whereas the leaves inoculated afterward, at 6 to 19 DAT, were not. 

The sporangial suspensions for artificial inoculation were prepared as follows. A *P. viticola* population, collected from different commercial vineyards and untreated plots located in Northern Italy between 2020 and 2022, was maintained in a glasshouse on potted plants of *V. vinifera* Merlot. Leaves with fresh DM lesions were cut from plants, enclosed in moistened polyethylene bags, and incubated in a growth chamber for 24 h at 20 °C, under a 12-h photoperiod, to stimulate the production of fresh sporangia. These sporangia were then gently removed from the lesions with a sterile cotton swab and put in distilled sterile water. Next, the sporangial concentration in the suspension was determined by a hemocytometer (BLAUBRAND Bürker-Türk, BRAND GMBH, Wertheim Germany) under an optical bright field microscope (40× magnification) and adjusted to a concentration of 1 × 10^4^ sporangia/mL [100]. 

### 4.4. Disease Assessment

Grapevine leaves were observed daily until the first signs of sporulation appeared on the foliage of the nontreated control. The inoculated leaves were then detached and brought to the laboratory in a cooler. Pictures of the upper blade of single leaves were taken with a digital camera; leaf size (in cm^2^) a nd disease severity (the percentage of leaf area showing typical DM symptoms; i.e., chlorotic ‘oil’ spots) were assessed using the software Asses (Assess 2.0; [101]). Leaves were then kept in a growth chamber for 24 h, at 20 °C and 100% relative humidity under a 12 h photoperiod, to promote the production of sporangia. Later, pictures of the lower leaf blade were taken and analyzed as before, to assess the sporulating severity (the percentage of leaf area showing typical DM sporulation in the form of white mold). 

### 4.5. Sporulation Assessment 

Using scissors, the collected individual leaves were then cut into pieces (ca. 1 cm^2^ in size). These were placed in a Becker with 5 mL of distilled water and vortexed for 5 min. The concentration of sporangia in the resulting suspension was determined in 10 μL drops with four repetitions under a microscope (40× magnification) by using a hemocytometer. The total numbers of sporangia per leaf and cm^2^ of the sporulating area were calculated, the latter as follows: sporangia per leaf × (leaf size in cm^2^/sporulating lesion size in cm^2^). 

### 4.6. Quantification of P. viticola DNA 

After the sporulation assessment, 10 random leaves per treatment were gently washed in sterile distilled water, with the help of a brush, to remove any remaining sporangiophores. Next, these eaves were disinfected with ethanol 70% for 30′ s, then washed again in distilled sterile water, and finally stored in a freezer (–20 °C) until the extraction of *P. viticola* DNA. 

Each DNA extraction was performed as described in Toffolatti et al. [38], albeit with slight modifications made as follows: leaf samples were cut into finer pieces (ca. 2 cm^2^ in size), put in a mortar, freeze-dried with liquid nitrogen, and ground into a fine powder that was inserted in sterile safe-lock 2 mL tubes (Eppendorf SE, Hamburg, Germany). Then, 100–150 mg of leaf sample powder was placed in a 2 mL microcentrifuge tube containing 650 μL of cetyltrimethylammonium bromide (CTAB) extraction buffer (2% CTAB, 100 mM Tris-HCl pH 8.0, 20 mM ethylenediaminetetraacetic acid [EDTA], 1.4 M NaCl, and 1% polyvinylpyrrolidone [PVP]), 100 mg of glass sand (425–600 μm in diameter), and two stainless steel beads (5 mm in diameter; Qiagen S.r.l., Milan, Italy). All tubes were then placed in a TissueLyser II (Qiagen, Italy), shaken for 1 min at 30 cycles/s, and placed on a heat block at 65 °C for 90 min. Total DNA was purified with chloroform-isoamyl alcohol (24:1) (*v*:*v*), precipitated with ice-cooled isopropanol, cleared with ethanol 70%, and resuspended in 40 μL of sterile ultrapure water. Both the yield and purity of the extracted DNA were determined by a NanoPhotometer^®^ N60 (Implen GmbH, Munich, Germany). The total DNA of each sample was finally adjusted to 20 ng/μL. 

To quantify the *P. viticola* DNA, we used two specific primer pairs and a hydrolysis probe (Giop) designed to target the internal transcribed spacer 1 (ITS 1)-5.8S rDNA. The *V. vinifera* DNA was also quantified to check that the amount of host plant DNA was constant in a given analyzed leaf sample [102]. Two specific primer pairs and a hydrolysis probe (Res) designed to target resveratrol synthase gene I were used. The (Giop) sequences were as follows: Giop F: 5′-TCC TGC AAT TCG CAT TAC GT-3′; Giop R: 5′-GGT TGC AGC TAA TGG ATT CCT A-3′; Giop P: 5′-6-FAM-TCG CAG TTC GCA GCG TTC TTC A-None-3′ with the fluorescent reporter FAM (6-carboxyfluorescein). The Res sequences were as follows: Res F: 5′-CGA GGA ATT TAG AAA CGC TCA AC-3′, Res R: 5′-GCT GTG CCA ATG GCT AGG A-3′, and Res P: 5′-HEX-TGC CAA GGG TCC GGC CAC C-BHQ2-3′ [102].

Both singleplex and duplex real-time PCR assays were conducted in an Applied Biosystems StepOnePlus™ System (Thermo Fisher Scientific Inc., Waltham, MA, USA). The thermocyling reaction conditions consisted of an initial incubation at 95 °C for 1 min followed by 40 cycles of 95 °C for 15 s and 60 °C for 30 s. Singleplex reaction mixtures contained 1× Luna Universal Probe qPCR Master Mix (New England Biolabs, Ipswich, UK), 250 nM of probes (GiopP or ResP), 900 nM of each of *P. viticola* forward and reverse primer (Giop F/R) or 120 nM of each of *V. vinifera* forward and reverse primer, and 2 μL of DNA template in a final volume of 10 μL. Duplex reaction mixtures contained the 1× Luna Universal Probe qPCR Master Mix, 250 nM of both *P. viticola* and *V. vinifera* probes (Giop P and Res P), 900 nM of each of *P. viticola* forward and reverse primer (Giop F/R), 120 nM of each of *V. vinifera* forward and reverse primer (Res F/R), and 2 μL of DNA template in a final volume of 10 μL. The sensitivity of the qPCR assays and the standard curve was determined as described in the Supplementary Material section. DNA amounts were finally obtained by transforming the quantification cycles (Cq) values of both targets (*P. viticola* and *V. vinifera*) as follows:

(1) $$DNA\;(ng/\mu L)\;=\;10^{\left[(Cq\;value\;-\;y\text{-}axis\;b)/a\right]}$$

where a = –3.4278 and b = 23.67 are the slope and intercept, respectively, of *P. viticola* DNA in the *V. vinifera* DNA standard curve (see Supplementary Materials). 

Given that the *V. vinifera* DNA was found to be mostly constant among the leaf samples—with an average concentration of 54.8 ng/µL and standard error of 3.6 ng/µL—we did not normalize the *P. viticola* DNA over the *V. vinifera* DNA [102]. The concentration of *P. viticola* DNA (ng/µL) was expressed on a per leaf basis and per cm^2^ of the affected area, the latter calculated as follows: DNA per leaf × (leaf size in cm^2^/DM lesion size in cm^2^). 

### 4.7. Data Analysis

Disease and sporulation severity data, as well as *P. viticola* DNA data (dependent variables), were subjected separately to an analysis of covariance (ANCOVA). This is a general linear model in which the experiment (three replicate experiments, considered as a random variable), treatments (six PRIs and the NT), DAT (1, 3, 6, 12, and 19 days after treatment), and their interactions were the sources of variance. Leaf size was used as a covariate because it is expected to share an essential amount of variance with the dependent variable and to also reduce error variance, thus increasing the power of the statistical test. The disease and sporulation severity data (both expressed as %) were arcsine transformed; the number of sporangia, ng of DNA, and sizes in cm^2^ were ln-transformed (natural logarithm function) prior to their ANCOVA. Means were compared by Fisher’s Protected least squares difference test with an alpha = 0.05. Averaged data were also expressed to convey the effect size in terms of the percentage reduction relative to the NT (nontreated) test group as follows: % reduction = [(*NT* − *T*)/*NT*] × 100 [103].

Finally, Pearson correlation coefficients (*r*) were calculated to test for linear relationships among disease and sporulation assessments and *P. viticola* DNA concentrations for the three datasets separately: that is, all the data pooled (treated and NT), as well as treated only, and nontreated only.

## Figures and Tables

**Table 1:** Experiment details, disease data for the nontreated grapevines, and weather data for the three conducted experiments.

Exp	Treatment with PRIs	DAT ^1^	*P. viticola* Inoculation	Sporulation Onset	N. of Days of Latency	Disease Severity (%)		Weather Data during Latency			
								T (°C)	RH (%)	LW (hours)	Rain (mm)
1	28 May 2020	1	29 May	5 June	7	36.6 ^2^	5.8 ^3^	19.0	63.8	35	29.6
		3	31 May	8 June	8	46.0	7.3	19.5	68.6	34	33.0
		6	3 June	15 June	12	21.7	4.6	18.9	77.6	58	63.0
		12	12 June	22 June	13	31.6	6.0	21.3	67.8	30	42.2
		19	16 June	28 June	12	12.2	2.1	23.4	60.4	21	22.8
2	7 July 2021	1	8 July	22 July	14	3.2	0.7	24.2	57.1	1	0.8
		3	10 July	27 July	17	4.2	1.7	24.3	60.1	2	4.4
		6	13 July	4 August	22	1.3	0.6	25.1	62.0	28	25.6
		12	19 July	6 August	18	3.3	2.6	25.6	62.8	28	25.6
		19	27 July	10 August	22	7.7	2.5	26.4	60.6	27	22.0
3	1 June 2022	1	2 June	14 June	12	11.1	2.6	25.0	55.2	0	0.0
		3	4 June	16 June	12	16.1	3.8	25.3	53.1	0	0.0
		6	7 June	21 June	14	4.7	0.9	24.8	54.1	31	13.9
		12	13 June	29 June	16	10.5	2.8	24.6	53.7	31	13.9
		19	20 June	11 July	20	6.8	1.4	24.8	55.8	31	18.5

^1^ Days After Treatment; ^2^ average disease severity in leaves of untreated plants; ^3^ standard errors. Temperature (T), Relative Humidity (RH), and Leaf Wetness (LW).

## Data Availability

The raw data supporting the conclusions of this article will be made available by the authors without undue reservation.

## Supplementary Materials

The following supporting information can be downloaded at: https://www.mdpi.com/article/10.3390/plants12162938/s1. Figure S1. Pearson correlations between leaf size (cm^2^) and different assessments of downy mildew (DM) in grapevine leaves collected from plants not treated and inoculated with *Plasmopara viticola* (Pv). Filled circles indicate means and the whiskers are standard errors; *r* is the Pearson correlation coefficient, and P is the probability level for the correlation. Table S1. Linear regression and reactions efficiency (E) for the relationship between serially diluted DNA concentrations (ln-transformed) of *P. viticola* or *V. vinifera,* and corresponding Cq values obtained in singleplex and duplex quantitative polymerase chain reaction (qPCR) assays. Figure S2. Standard curves of *P.viticola* DNA diluted in *V. vinifera* DNA, *P.viticola* and *V.vinifera* DNA diluted in water. Quantification cycles (Cq) were plotted against the log (DNA) standards of known concentrations. Each data point represents the average of three replications. Ref. [104] is cited in Supplementary Materials.

## References

[1] Koledenkova K., Esmaeel Q., Jacquard C., Nowak J., Clément C., Barka E.A. (2022). *Plasmopara viticola* the causal agent of downy mildew of grapevine: From its taxonomy to disease management. Front. Microbiol..

[2] Kim K.-H., Kabir E., Jahan S.A. (2017). Exposure to pesticides and the associated human health effects. Sci. Total Environ..

[3] Sandroni M., Liljeroth E., Mulugeta T., Alexandersson E. (2020). Plant resistance inducers (PRIs): Perspectives for future disease management in the field. CAB Rev..

[4] Delaunois B., Farace G., Jeandet P., Clément C., Baillieul F., Dorey S., Cordelier S. (2014). Elicitors as alternative strategy to pesticides in grapevine? Current knowledge on their mode of action from controlled conditions to vineyard. Environ. Sci. Pollut. Res..

[5] Jones J.D., Dangl J.L. (2006). The plant immune system. Nature.

[6] Gomès E., Coutos-Thévenot P. (2009). Molecular aspects of grapevine-pathogenic fungi interactions. Grapevine Molecular Physiology & Biotechnology.

[7] Wilkinson S.W., Magerøy M.H., López Sánchez A., Smith L.M., Furci L., Cotton T.A., Krokene P., Ton J. (2019). Surviving in a hostile world: Plant strategies to resist pests and diseases. Annu. Rev. Phytopathol..

[8] Bigeard J., Colcombet J., Hirt H. (2015). Signaling mechanisms in pattern-triggered immunity (PTI). Mol. Plant.

[9] Yu X., Feng B., He P., Shan L. (2017). From chaos to harmony: Responses and signaling upon microbial pattern recognition. Annu. Rev. Phytopathol..

[10] Héloir M.-C., Adrian M., Brulé D., Claverie J., Cordelier S., Daire X., Dorey S., Gauthier A., Lemaître-Guillier C., Negrel J. (2019). Recognition of elicitors in grapevine: From MAMP and DAMP perception to induced resistance. Front. Plant Sci..

[11] Urban L., Lauri F., Ben Hdech D., Aarrouf J. (2022). Prospects for Increasing the Efficacy of Plant Resistance Inducers Stimulating Salicylic Acid. Agronomy.

[12] Harm A., Kassemeyer H.-H., Seibicke T., Regner F. (2011). Evaluation of chemical and natural resistance inducers against downy mildew (*Plasmopara viticola*) in grapevine. Am. J. Enol. Vitic..

[13] Gutiérrez--Gamboa G., Romanazzi G., Garde--Cerdán T., Pérez--Álvarez E.P. (2019). A review of the use of biostimulants in the vineyard for improved grape and wine quality: Effects on prevention of grapevine diseases. J. Sci. Food Agric..

[14] Héloir M.-C., Khiook I.L.K., Lemaître-Guillier C., Clément G., Jacquens L., Bernaud E., Trouvelot S., Adrian M. (2018). Assessment of the impact of PS3-induced resistance to downy mildew on grapevine physiology. Plant Physiol. Biochem..

[15] Aziz A., Poinssot B., Daire X., Adrian M., Bézier A., Lambert B., Joubert J.-M., Pugin A. (2003). Laminarin elicits defense responses in grapevine and induces protection against Botrytis cinerea and *Plasmopara viticola*. Mol. Plant-Microbe Interact..

[16] Rinaudo M. (2006). Chitin and chitosan: Properties and applications. Prog. Polym. Sci..

[17] van Aubel G., Buonatesta R., Van Cutsem P. (2014). COS-OGA: A novel oligosaccharidic elicitor that protects grapes and cucumbers against powdery mildew. Crop Prot..

[18] Pujos P., Martin A., Farabullini F., Pizzi M. RomeoTM, cerevisane-based biofungicide against the main diseases of grape and of other crops: General description. Proceedings of the Atti, Giornate Fitopatologiche, Chianciano Terme.

[19] Mohamed N., Lherminier J., Farmer M.-J., Fromentin J., Béno N., Houot V., Milat M.-L., Blein J.-P. (2007). Defense responses in grapevine leaves against Botrytis cinerea induced by application of a Pythium oligandrum strain or its elicitin, oligandrin, to roots. Phytopathology.

[20] Gerbore J., Bruez E., Vallance J., Grizard D., Regnault-Roger C., Rey P. (2016). Protection of grapevines by Pythium oligandrum strains isolated from Bordeaux vineyards against powdery mildew. Biocontrol of Major Grapevine Diseases: Leading Research.

[21] Rekanović E., Potočnik I., Stepanović M., Milijašević S., Todorović B. (2008). Field efficacy of fluopicolide and fosetyl-Al fungicide combination (Profiler^®^) for control of *Plasmopara viticola* (Berk. & Curt.) Berl. & Toni. in grapevine. Pestic. I Fitomedicina.

[22] Lim S., Borza T., Peters R.D., Coffin R.H., Al-Mughrabi K.I., Pinto D.M., Wang-Pruski G. (2013). Proteomics analysis suggests broad functional changes in potato leaves triggered by phosphites and a complex indirect mode of action against Phytophthora infestans. J. Proteom..

[23] Rienth M., Crovadore J., Ghaffari S., Lefort F. (2019). Oregano essential oil vapour prevents *Plasmopara viticola* infection in grapevine (Vitis Vinifera) and primes plant immunity mechanisms. PLoS ONE.

[24] Lakkis S., Trotel-Aziz P., Rabenoelina F., Schwarzenberg A., Nguema-Ona E., Clément C., Aziz A. (2019). Strengthening grapevine resistance by Pseudomonas fluorescens PTA-CT2 relies on distinct defense pathways in susceptible and partially resistant genotypes to downy mildew and gray mold diseases. Front. Plant Sci..

[25] Pezzotti G., Fujita Y., Boschetto F., Zhu W., Marin E., Vandelle E., McEntire B.J., Bal S.B., Giarola M., Makimura K. (2020). Activity and Mechanism of Action of the Bioceramic Silicon Nitride as an Environmentally Friendly Alternative for the Control of the Grapevine Downy Mildew Pathogen *Plasmopara viticola*. Front. Microbiol..

[26] Marcianò D., Ricciardi V., Fassolo E.M., Passera A., Bianco P.A., Failla O., Casati P., Maddalena G., De Lorenzis G., Toffolatti S.L. (2021). RNAi of a putative grapevine susceptibility gene as a possible downy mildew control strategy. Front. Plant Sci..

[27] Taillis D., Pébarthé-Courrouilh A., Lepeltier É., Petit E., Palos-Pinto A., Gabaston J., Mérillon J.-M., Richard T., Cluzet S. (2022). A grapevine by-product extract enriched in oligomerised stilbenes to control downy mildews: Focus on its modes of action towards *Plasmopara viticola*. OENO One.

[28] Krzyzaniak Y., Trouvelot S., Negrel J., Cluzet S., Valls J., Richard T., Bougaud A., Jacquens L., Klinguer A., Chiltz A. (2018). A plant extract acts both as a resistance inducer and an oomycide against grapevine downy mildew. Front. Plant Sci..

[29] Cappelletti M., Perazzolli M., Antonielli L., Nesler A., Torboli E., Bianchedi P.L., Pindo M., Puopolo G., Pertot I. (2016). Leaf treatments with a protein-based resistance inducer partially modify phyllosphere microbial communities of grapevine. Front. Plant Sci..

[30] Boubakri H., Wahab M.A., Chong J., Bertsch C., Mliki A., Soustre-Gacougnolle I. (2012). Thiamine induced resistance to *Plasmopara viticola* in grapevine and elicited host–defense responses, including HR like-cell death. Plant Physiol. Biochem..

[31] Trouvelot S., Varnier A.-L., Allegre M., Mercier L., Baillieul F., Arnould C., Gianinazzi-Pearson V., Klarzynski O., Joubert J.-M., Pugin A. (2008). A β-1, 3 glucan sulfate induces resistance in grapevine against *Plasmopara viticola* through priming of defense responses, including HR-like cell death. Mol. Plant-Microbe Interact..

[32] Bleyer G., Lösch F., Schumacher S., Fuchs R. (2020). Together for the Better: Improvement of a Model Based Strategy for Grapevine Downy Mildew Control by Addition of Potassium Phosphonates. Plants.

[33] Romanazzi G., Mancini V., Feliziani E., Servili A., Endeshaw S., Neri D. (2016). Impact of alternative fungicides on grape downy mildew control and vine growth and development. Plant Dis..

[34] Calderone F., Vitale A., Panebianco S., Lombardo M.F., Cirvilleri G. (2022). COS-OGA Applications in Organic Vineyard Manage Major Airborne Diseases and Maintain Postharvest Quality of Wine Grapes. Plants.

[35] Garde-Cerdán T., Mancini V., Carrasco-Quiroz M., Servili A., Gutiérrez-Gamboa G., Foglia R., Pérez-Álvarez E.P., Romanazzi G. (2017). Chitosan and laminarin as alternatives to copper for *Plasmopara viticola* control: Effect on grape amino acid. J. Agric. Food Chem..

[36] Parlevliet J.E. (1979). Components of resistance that reduce the rate of epidemic development. Annu. Rev. Phytopathol..

[37] Bove F., Bavaresco L., Caffi T., Rossi V. (2019). Assessment of resistance components for improved phenotyping of grapevine varieties resistant to downy mildew. Front. Plant Sci..

[38] Toffolatti S.L., Venturini G., Maffi D., Vercesi A. (2012). Phenotypic and histochemical traits of the interaction between *Plasmopara viticola* and resistant or susceptible grapevine varieties. BMC Plant Biol..

[39] Salotti I., Bove F., Ji T., Rossi V. (2022). Information on disease resistance patterns of grape varieties may improve disease management. Front. Plant Sci..

[40] Bove F., Rossi V. (2020). Components of partial resistance to *Plasmopara viticola* enable complete phenotypic characterization of grapevine varieties. Sci. Rep..

[41] EPPO (2020). EPPO Standards PP1, Efficacy Evaluation of Plant Protection Products, PP1/31 (3)—Plasmopara viticola.

[42] Gadoury D.M., Seem R.C., Wilcox W.F., Kennelly M.M., Magarey P.A., Dry I.B., Gubler W., Pscheidt J.W., Grove G., Sutton T.B. Modeling and mapping the relationship between climate and ontogenic resistance to the major fungal diseases of grapevine. Proceedings of the 5th International Workshop on Grapevine Downy and Powdery Mildew.

[43] Kennelly M.M., Gadoury D.M., Wilcox W.F., Magarey P.A., Seem R.C. (2005). Seasonal development of ontogenic resistance to downy mildew in grape berries and rachises. Phytopathology.

[44] Mertes C., Schumacher S., Kaltenbach T., Bleyer G., Fuchs R. (2022). Studies on the resistance of different developmental stages in susceptible and tolerant grapevine c *Plasmopara viticola*. BIO Web of Conferences.

[45] Bleyer G., Huber B., Steinmetz V., Kassemeyer H. (2003). Growth-models, a tool to define spray intervals against downy mildew (*Plasmopara viticola*). IOBC Wprs Bull..

[46] De Miccolis Angelini R.M., Rotolo C., Gerin D., Abate D., Pollastro S., Faretra F. (2019). Global transcriptome analysis and differentially expressed genes in grapevine after application of the yeast—Derived defense inducer cerevisane. Pest Manag. Sci..

[47] Dufour M.-C., Corio-Costet M.-F. (2013). Variability in the sensitivity of biotrophic grapevine pathogens (Erysiphe necator and *Plasmopara viticola*) to acibenzolar-S methyl and two phosphonates. Eur. J. Plant Pathol..

[48] Walters D., Fountaine J. (2009). Practical application of induced resistance to plant diseases: An appraisal of effectiveness under field conditions. J. Agric. Sci..

[49] Gauthier A., Trouvelot S., Kelloniemi J., Frettinger P., Wendehenne D., Daire X., Joubert J.-M., Ferrarini A., Delledonne M., Flors V. (2014). The sulfated laminarin triggers a stress transcriptome before priming the SA-and ROS-dependent defenses during grapevine’s induced resistance against *Plasmopara viticola*. PLoS ONE.

[50] Kamle M., Borah R., Bora H., Jaiswal A.K., Singh R.K., Kumar P. (2020). Systemic acquired resistance (SAR) and induced systemic resistance (ISR): Role and mechanism of action against phytopathogens. Fungal Biotechnol. Bioeng..

[51] van Aubel G., Cambier P., Dieu M., Van Cutsem P. (2016). Plant immunity induced by COS-OGA elicitor is a cumulative process that involves salicylic acid. Plant Sci..

[52] Aziz A., Trotel-Aziz P., Dhuicq L., Jeandet P., Couderchet M., Vernet G. (2006). Chitosan oligomers and copper sulfate induce grapevine defense reactions and resistance to gray mold and downy mildew. Phytopathology.

[53] Huot B., Yao J., Montgomery B.L., He S.Y. (2014). Growth–defense tradeoffs in plants: A balancing act to optimize fitness. Mol. Plant.

[54] Dufour M.-C., Druelle L., Sauris P., Taris G., Corio-Costet M.-F. (2009). Impact of grapevine downy and powdery mildew diversity on the efficacy of phosphonate derivatives (fosétyl-Al, PK2) described like stimulator of plant defences. Proceedings of the 9 ème Conférence International sur les Maladies des Plantes.

[55] Liljeroth E., Lankinen Å., Wiik L., Burra D.D., Alexandersson E., Andreasson E. (2016). Potassium phosphite combined with reduced doses of fungicides provides efficient protection against potato late blight in large-scale field trials. Crop Prot..

[56] Nelson T., Lewis B. (1974). Separation and characterization of the soluble and insoluble components of insoluble laminaran. Carbohydr. Res..

[57] Allègre M., Héloir M.-C., Trouvelot S., Daire X., Pugin A., Wendehenne D., Adrian M. (2009). Are grapevine stomata involved in the elicitor-induced protection against downy mildew?. Mol. Plant-Microbe Interact..

[58] Balestrini R., Ghignone S., Quiroga G., Fiorilli V., Romano I., Gambino G. (2020). Long-term impact of chemical and alternative fungicides applied to Grapevine cv Nebbiolo on Berry Transcriptome. Int. J. Mol. Sci..

[59] Paris F., Krzyżaniak Y., Gauvrit C., Jamois F., Domergue F., Joubès J., Ferrières V., Adrian M., Legentil L., Daire X. (2016). An ethoxylated surfactant enhances the penetration of the sulfated laminarin through leaf cuticle and stomata, leading to increased induced resistance against grapevine downy mildew. Physiol. Plant..

[60] Pugliese M., Monchiero M., Gullino M.L., Garibaldi A. (2018). Application of laminarin and calcium oxide for the control of grape powdery mildew on Vitis vinifera cv. Moscato. J. Plant Dis. Prot..

[61] Hossain M.A., Liu F., Burrit D.J., Fujita M., Huang B. (2020). Priming-Mediated Stress and Cross-Stress Tolerance in Crop Plants.

[62] Koçi R., Dupuy F., Lebbar S., Gloaguen V., Faugeron Girard C. (2022). A New Promising Plant Defense Stimulator Derived from a By-Product of Agar Extraction from Gelidium sesquipedale. Horticulturae.

[63] Steimetz E., Trouvelot S., Gindro K., Bordier A., Poinssot B., Adrian M., Daire X. (2012). Influence of leaf age on induced resistance in grapevine against *Plasmopara viticola*. Physiol. Mol. Plant Pathol..

[64] Lee S., Choi H., Suh S., Doo I.-S., Oh K.-Y., Jeong Choi E., Schroeder Taylor A.T., Low P.S., Lee Y. (1999). Oligogalacturonic acid and chitosan reduce stomatal aperture by inducing the evolution of reactive oxygen species from guard cells of tomato and Commelina communis. Plant Physiol..

[65] de Borba M.C., Velho A.C., de Freitas M.B., Holvoet M., Maia-Grondard A., Baltenweck R., Magnin-Robert M., Randoux B., Hilbert J.-L., Reignault P. (2022). A laminarin-based formulation protects wheat against Zymoseptoria tritici via direct antifungal activity and elicitation of host defense-related genes. Plant Dis..

[66] Besrukow P., Will F., Dussling S., Berkelmann-Löhnertz B., Schweiggert R. (2023). Additive and Synergistic Antifungal Effects of Copper and Phenolic Extracts from Grape Cane and Apples. Pest Manag. Sci..

[67] Mestre P., Arista G., Piron M.C., Rustenholz C., Ritzenthaler C., Merdinoglu D., Chich J.F. (2017). Identification of a Vitis vinifera endo-β-1, 3-glucanase with antimicrobial activity against *Plasmopara viticola*. Mol. Plant Pathol..

[68] El Hadrami A. (2010). Adam LR El Hadrami I. Daayf F. Chitosan in plant protection. Mar. Drugs.

[69] Tröster V., Setzer T., Hirth T., Pecina A., Kortekamp A., Nick P. (2017). Probing the contractile vacuole as Achilles’ heel of the biotrophic grapevine pathogen *Plasmopara viticola*. Protoplasma.

[70] Manghi M.C., Masiol M., Calzavara R., Graziano P.L., Peruzzi E., Pavoni B. (2021). The use of phosphonates in agriculture. Chemical, biological properties and legislative issues. Chemosphere.

[71] Magarey P., Wachtel M., Newton M. (1991). Evaluation of phosphonate, fosetyl-Al and several phenylamide fungicides for post-infection control of grapevine downy mildew caused by *Plasmopara viticola*. Australas. Plant Pathol..

[72] Pinto K.M.S., do Nascimento L.C., de Souza Gomes E.C., da Silva H.F., dos Reis Miranda J. (2012). Efficiency of resistance elicitors in the management of grapevine downy mildew *Plasmopara viticola*: Epidemiological, biochemical and economic aspects. Eur. J. Plant Pathol..

[73] Dufour M.-C., Magnin N., Dumas B., Vergnes S., Corio-Costet M.-F. (2016). High-throughput gene-expression quantification of grapevine defense responses in the field using microfluidic dynamic arrays. BMC Genom..

[74] Guest D., Grant B. (1991). The complex action of phosphonates as antifungal agents. Biol. Rev..

[75] Fenn M., Coffey M. (1984). Studies on the in vitro and in vivo antifungal activity of fosetyl-Al and phosphorous acid. Phytopathology.

[76] Magarey P., Wicks T., Wachtel M. (1990). Phosphonic (phosphorous) acid controls *Plasmopara viticola* the cause of downy mildew of grapevines. Australas. Plant Pathol..

[77] Gisi U. (2002). Chemical control of downy mildews. Advances in Downy Mildew Research.

[78] Wicks T., Magarey P., Wachtel M., Frensham A. (1991). Effect of postinfection application of phosphorous (phosphonic) acid on the incidence and sporulation of *Plasmopara viticola* on grapevine. Plant Dis..

[79] Mishko A., Lutsky E. (2020). The effect of Saccharomyces cerevisiae on antioxidant system of grape leaves infected by downy mildew. BIO Web of Conferences.

[80] Lopes M.R., Klein M.N., Ferraz L.P., da Silva A.C., Kupper K.C. (2015). Saccharomyces cerevisiae: A novel and efficient biological control agent for Colletotrichum acutatum during pre-harvest. Microbiol. Res..

[81] Singh R.R., Chinnasri B., De Smet L., Haeck A., Demeestere K., Van Cutsem P., Van Aubel G., Gheysen G., Kyndt T. (2019). Systemic defense activation by COS-OGA in rice against root-knot nematodes depends on stimulation of the phenylpropanoid pathway. Plant Physiol. Biochem..

[82] Clinckemaillie A., Decroës A., van Aubel G., Carrola dos Santos S., Renard M.E., Van Cutsem P., Legrève A. (2017). The novel elicitor COS-OGA enhances potato resistance to late blight. Plant Pathol..

[83] van Aubel G., Serderidis S., Ivens J., Clinckemaillie A., Legrève A., Hause B., Van Cutsem P. (2018). Oligosaccharides successfully thwart hijacking of the salicylic acid pathway by Phytophthora infestans in potato leaves. Plant Pathol..

[84] Bi Q., Han X., Ma Z., Zhao J., Jia H., Wang W. (2018). Inhibitory effect of Pythium oligandrum interaction with dimethomorph and the application of chemical decrement on grape downy mildew. Acta Phytopathol. Sin..

[85] Gerbore J., Benhamou N., Vallance J., Le Floch G., Grizard D., Regnault-Roger C., Rey P. (2014). Biological control of plant pathogens: Advantages and limitations seen through the case study of Pythium oligandrum. Environ. Sci. Pollut. Res..

[86] Bělonožníková K., Hýsková V., Chmelík J., Kavan D., Čeřovská N., Ryšlavá H. (2022). Pythium oligandrum in plant protection and growth promotion: Secretion of hydrolytic enzymes, elicitors and tryptamine as auxin precursor. Microbiol. Res..

[87] Plank J. (1963). Plant Diseases-Epidemics and Control.

[88] Bove F., Savary S., Willocquet L., Rossi V. (2021). Modelling the effect of partial resistance on epidemics of downy mildew of grapevine. Eur. J. Plant Pathol..

[89] Rossi V., Salinari F., Poni S., Caffi T., Bettati T. (2014). Addressing the implementation problem in agricultural decision support systems: The example of vite. net^®^. Comput. Electron. Agric..

[90] Rossi V., Caffi T., Giosuè S., Bugiani R. (2008). A mechanistic model simulating primary infections of downy mildew in grapevine. Ecol. Model..

[91] Brischetto C., Bove F., Fedele G., Rossi V. (2021). A Weather-Driven Model for Predicting Infections of Grapevines by Sporangia of *Plasmopara viticola*. Front. Plant Sci..

[92] Taibi O., Bardelloni V., Bove F., Scaglia F., Caffi T., Rossi V. (2022). Activity of resistance inducers against *Plasmopara viticola* in vineyard. BIO Web of Conferences.

[93] Nogueira Júnior A.F., Tränkner M., Ribeiro R.V., Von Tiedemann A., Amorim L. (2020). Photosynthetic cost associated with induced defense to *Plasmopara viticola* in grapevine. Front. Plant Sci..

[94] Conrath U., Beckers G.J., Langenbach C.J., Jaskiewicz M.R. (2015). Priming for enhanced defense. Annu. Rev. Phytopathol..

[95] Martinez-Medina A., Flors V., Heil M., Mauch-Mani B., Pieterse C.M., Pozo M.J., Ton J., van Dam N.M., Conrath U. (2016). Recognizing plant defense priming. Trends Plant Sci..

[96] Rossi V., Caffi T. (2007). Effect of water on germination of *Plasmopara viticola* oospores. Plant Pathol..

[97] Lorenz D.H., Eichhorn K.W., Bleiholder H., Klose R., Meier U., Weber E. (1995). Growth Stages of the Grapevine: Phenological growth stages of the grapevine (*Vitis vinifera* L. ssp. vinifera)-Codes and descriptions according to the extended BBCH scale. Aust. J. Grape Wine Res..

[98] Kennelly M.M., Gadoury D.M., Wilcox W.F., Magarey P.A., Seem R.C. (2007). Primary infection, lesion productivity, and survival of sporangia in the grapevine downy mildew pathogen *Plasmopara viticola*. Phytopathology.

[99] Blaeser M., Weltzien H. (1977). Untersuchungen über die Infektion von Weinreben mit *Plasmopara viticola* in Abhängigkeit von der Blattnässedauer. Meded. Fac. Landbouww. Rijksuniv. Gent.

[100] Caffi T., Gilardi G., Monchiero M., Rossi V. (2013). Production and release of asexual sporangia in *Plasmopara viticola*. Phytopathology.

[101] Lamari L. (2009). Assess: Image Analysis Software for Plant Disease Quantification V2.0.

[102] Valsesia G., Gobbin D., Patocchi A., Vecchione A., Pertot I., Gessler C. (2005). Development of a high-throughput method for quantification of *Plasmopara viticola* DNA in grapevine leaves by means of quantitative real-time polymerase chain reaction. Phytopathology.

[103] Abbott W.S. (1925). A method of computing the effectiveness of an insecticide. J. Econ. Entomol.

[104] Bustin S.A., Benes V., Garson J.A., Hellemans J., Huggett J., Kubista M., Mueller R., Nolan T., Pfaffl M.W., Shipley G.L. (2009). The MIQE guidelines: Minimum information for publication of quantitative real-time PCR experiments. Clin. Chem..

