# Supplementary material for: Plant Resistance Inducers Affect Multiple Epidemiological Components of Plasmopara viticola on Grapevine Leaves
Source: Plants (Basel). 2023 Aug 14;12(16):2938. doi: 10.3390/plants12162938 (PMC10459891; doi:10.3390/plants12162938)
Supplement: Supplementary file 1 [file plants-12-02938-s001.zip › plants-2540569-supplementary.pdf]

<u>Supplementary material</u>

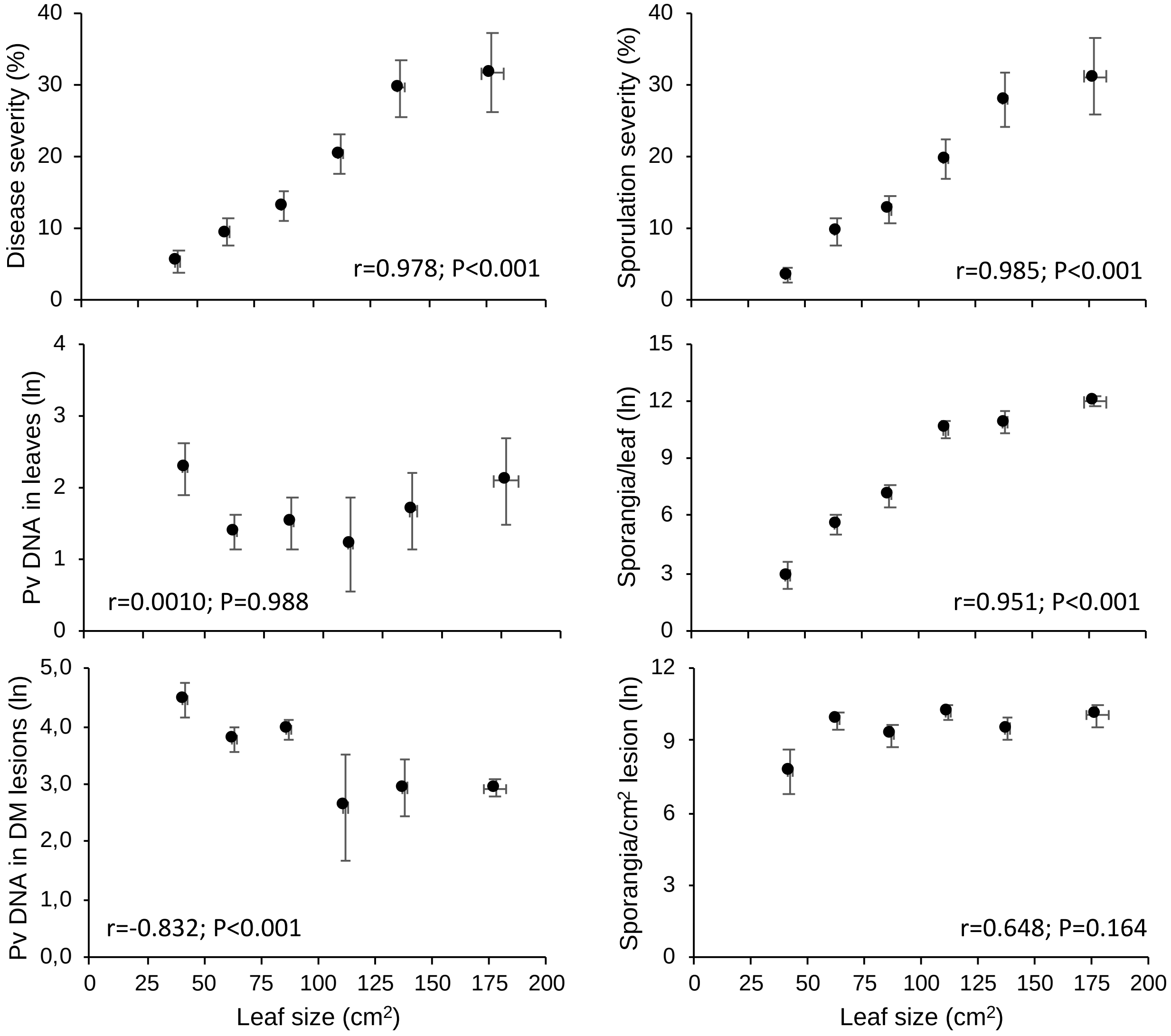

Figure S1. Pearson correlations between leaf size (cm^2) and different assessments of downy mildew (DM) in grapevine leaves collected from plants that have not been treated and inoculated with *Plasmopara viticola* (Pv). Filled circles indicate means and the whiskers are standard errors; *r* is the Pearson correlation coefficient, and P is the probability level for the correlation.

Sporangia of *P. viticola* were collected from an NT leaf with fresh sporulation. Then, they were suspended in sterile distilled water and centrifugated at 14,000 rpm for 10 min. The aqueous phase was discarded, and the sporangia in the pellet were stored at −20°C likewise the leaves samples until they were used for extraction of genomic DNA.

The sensitivity of the qPCR assay was assessed following an absolute quantification approach. Standard curves were obtained from two singleplex qPCR assays: the Giop assay with DNA of *P.viticola* from sporangia solution as a template in a 10-fold dilution series (from 10 to 0.0001 ng/μL), and the Res assay with DNA of *V.vinifera* extracted from fresh and healthy grapevine leaves as a template in a 10-fold dilution series (from 20 to 0.002 ng/μL). duplex qPCR assays were performed with DNA of *P.viticola* mixed with DNA of *V. vinifera*, following the approach described by [102] with slight modifications. In brief, 1μl from each of the previously described DNA dilutions for *P.viticola* was mixed with 1μL of *V. vinifera* DNA (20 ng/μL), yielding a 10-fold dilution series from 1:2 to 1:20,000 w/w of *P.viticola*/*V. vinifera* DNA. qPCR assays were carried out three times with the same DNA templates, each dilution was replicated three times too. Sterile distilled water control was included in triplicate in each assay. Standard curves of the qPCR assay were produced by linear regression, and the coefficient of determination (R^2) was calculated. The amplification efficiency (E) was determined from the slope of the standard curves [104].

Table S1. Linear regression and reactions efficiency (E) for the relationship between serially diluted DNA concentrations (ln-transformed) of *P. viticola* or *V. vinifera* and corresponding Cq values obtained in singleplex and duplex quantitative polymerase chain reaction (qPCR) assays.

qPCR assay	DNA template	Linear equation [a]	R^2 [b]	P value [c]	E (%)
Singleplex Giop	*P.viticola*	y = -3.4373x + 21.899	0.99	<0.001	95 %
Singleplex Res	*V.vinifera*	y = -3.2958x + 26.352	0.99	<0.001	101%
Duplex Giop/Res	*P.viticola* + *V.vinifera*	y = -3.4278x + 23.67	0.98	<0.001	96%

[a] in the equation, y refers to the Cq value, and x refers to the DNA concentration (ln-transformed). [B] R^2 is the coefficient of determination of regression. [C] P value indicates the tests of the fit of the regression model.

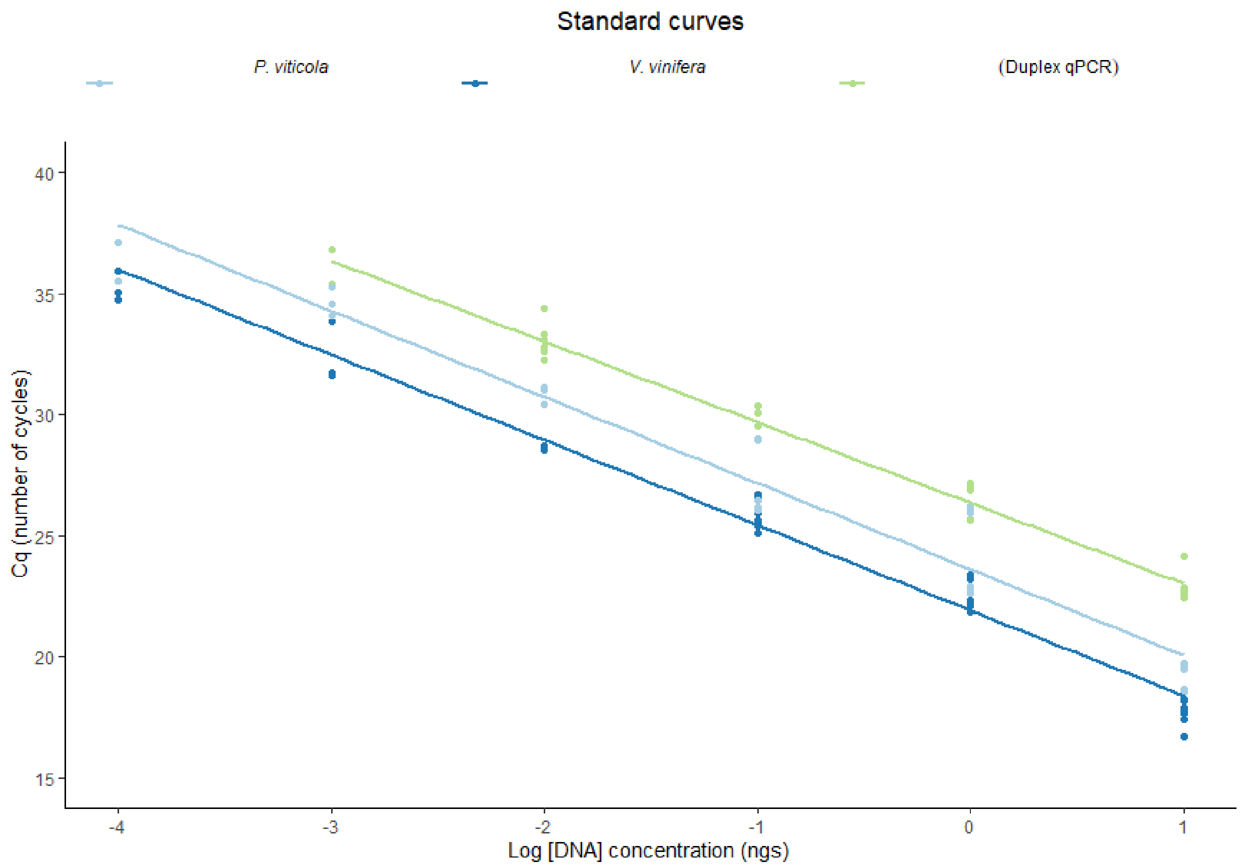

Figure S2. Standard curves of *P.viticola* DNA diluted in *V. vinifera* DNA , *P.viticola* and *V.vinifera* DNA diluted in water. Quantification cycles (Cq) were plotted against the log (DNA) standards of known concentrations. Each data point represents the average of three replications.
